# Comparative analysis of stereotactic radiosurgery and microsurgery for vestibular schwannoma: a clinical and radiological evaluation

**DOI:** 10.1007/s11060-026-05707-z

**Published:** 2026-07-18

**Authors:** Andre E. Boyke, Tariq Al-Saadi, Simon A. Menaker, Edward Robinson, Alan Nguyen, Michelot Michel, Brittany Morris, Younis Al-Mufargi, Behrooz Hakimian, Amin Mirhadi, Yu-Tung Wong, Mia E. Miller, John S. Yu

**Affiliations:** 1 Department of Neurosurgery, Cedars-Sinai Medical Center, Los Angeles, CA USA; 2 Morehouse School of Medicine, Atlanta, GA USA; 3 College of Medicine, Charles R. Drew University of Medicine and Science, Los Angeles, CA USA; 4 Department of Neurosurgery, Perelman School of Medicine, University of Pennsylvania, Philadelphia, PA USA; 5 Chicago Medical School at Rosalind Franklin University, North Chicago, IL USA; 6 Department of Surgery, Medical City for Military and Security Services, Muscat, Oman; 7 Department of Radiation Oncology, Cedars-Sinai Medical Center, Los Angeles, CA USA; 8 Division of Otolaryngology-Head and Neck Surgery, Cedars-Sinai Medical Center, Los Angeles, CA USA

**Keywords:** Vestibular schwannoma, Stereotactic radiosurgery, Microsurgery, Outcomes, Tumor control, Hearing preservation

## Abstract

**Purpose:**

To compare radiographic and clinical outcomes of vestibular schwannoma management using stereotactic radiosurgery (SRS) and microsurgical resection, with emphasis on paired pre- and post-treatment changes in tumor size and patient outcomes.

**Methods:**

A retrospective review was conducted of 87 patients treated for vestibular schwannoma between 2013 and 2024 at a single tertiary center. Demographic, clinical, and radiographic variables, including hearing loss, facial weakness, brainstem compression, tumor area, and Koos grade, were analyzed. Tumor area was determined from radiology reports and compared using nonparametric statistical tests, with significance defined as *p* < 0.05.

**Results:**

Microsurgery achieved a median 76% reduction in tumor area, while SRS was associated with relative stability or modest growth (+ 5%) (*p* < 0.001). Post-treatment rates of hearing loss and facial weakness were similar between groups, suggesting that substantial tumor reduction following microsurgery was not associated with increased morbidity in this cohort. Koos grade correlated with both brainstem compression and hearing loss, with higher grades favoring surgical management (*p* = 0.049). In the Koos IV subset, surgery achieved significant tumor reduction without disproportionate postoperative deficits compared with lower grades.

**Conclusion:**

Microsurgery provides immediate and substantial tumor reduction without increased morbidity, while SRS maintains radiographic stability with favorable clinical outcomes. These findings underscore the complementary roles of both modalities and support the Koos classification as a practical and reliable framework for treatment selection.

## Introduction

Vestibular schwannomas (VS), also known as acoustic neuromas, are benign tumors that arise from Schwann cells of the vestibular division of the eighth cranial nerve and are typically located in the cerebellopontine angle [1, 2]. These lesions are generally slow growing, with reported average growth rates of approximately 1–2 mm per year [1, 3]. Given their proximity to critical neurovascular structures, VS can cause significant morbidity. Clinical presentations most commonly include progressive unilateral sensorineural hearing loss, tinnitus, imbalance, and, in larger tumors, brainstem compression with cranial nerve neuropathies [4, 5]. 

Current treatment options include observation, stereotactic radiosurgery (SRS), and microsurgical resection. Each of these approaches has a distinct risk–benefit profile, depending on patient age, presenting symptoms, tumor size, and the risk of anatomical or symptomatic progression [6, 7]. For large tumors (commonly greater than 2.5–3 cm in maximum diameter), or those associated with brainstem compression or disabling symptoms, microsurgical resection remains the standard of care. The goals of surgery include tumor debulking and histopathologic confirmation, with functional outcomes, particularly hearing preservation, largely dependent on preoperative auditory status and the choice of surgical approach (retrosigmoid, middle fossa, or translabyrinthine) [2, 5, 6, 8]. 

In contrast, SRS has gained increasing use for its minimally invasive profile and high long-term tumor control rates in small to medium-sized lesions (< 2–3 cm). Tumor shrinkage after SRS may be delayed, and treatment carries risks of radiation-induced cranial neuropathy or, rarely, malignant transformation [9, 10]. Despite widespread adoption of both modalities, comparative data remain limited, especially regarding volumetric tumor response, cranial nerve outcomes, and the impact of tumor characteristics such as Koos grade or morphology on efficacy [6, 11]. Real-world treatment decisions are frequently influenced by institutional expertise and philosophy, rather than uniform evidence-based criteria.

In this retrospective cohort study, we compare clinical and radiological outcomes of patients with vestibular schwannomas treated with either SRS or microsurgery at our institution over an eleven-year period (2013–2024). Using paired pre- and post-treatment data, we describe tumor response, facial and hearing function, and their relationship with preoperative variables such as age, Koos grade, and brainstem compression. Our goal is to develop a data-driven framework that guides individualized treatment planning in this heterogeneous patient population.

## Methods

The medical records and radiographs of patients treated for neurogenic tumors at our center from 2013 to 2024 were retrospectively reviewed. Patients diagnosed with tumors other than schwannoma based on histopathological examination were excluded from the study. The parameters collected included patient age, gender, pre- and post-operative clinical symptoms (hearing loss, facial weakness), radiographic features (tumor size, brainstem compression, tumor morphology, tumor status, Koos score), and treatment type (stereotactic radiosurgery vs. microsurgery). Tumor size was recorded as area (cm²) based on radiology reports, as volumetric measurements were not consistently available across all patients.

Treatment allocation was determined based on tumor characteristics (including size and Koos grade), presence of brainstem compression, patient clinical status, multidisciplinary discussion, and patient preference.

All analyses were performed using IBM SPSS Statistics (Version 28), employing appropriate statistical tests such as the Student’s t-test, Mann–Whitney U test, and Wilcoxon signed-rank test for non-normally distributed variables, the Shapiro–Wilk test for normality assessment, and the chi-square test for categorical variables. A p-value < 0.05 was considered statistically significant. Due to the retrospective design’s inherent variability in documentation, detailed data on stereotactic radiosurgery parameters (including dose and fractionation), as well as standardized follow-up intervals and regrowth rates following subtotal resection, were not consistently available across all patients. Differences in sample size across analyses reflect the availability of paired pre- and post-treatment imaging. Percentage change calculations were restricted to patients with complete paired measurements. Missing data were due to incomplete imaging records or non-standardized follow-up intervals inherent to retrospective data collection. Therefore, analysis was restricted to variables that were uniformly reported within the cohort.

## Results

### **Demographic and **clinical characteristics

Stereotactic radiosurgery (SRS) patients and microsurgery patients underwent vestibular schwannoma treatment according to the clinical parameters in Table 1. The mean age of the patients was 55.87 ± 14.71 years and SRS patients were older than microsurgery patients by 59.47 ± 13.58 years compared to 52.36 ± 15.11 years (*p* = 0.037). Age was not normally distributed (Shapiro–Wilk *p* = 0.018) so the Mann–Whitney U test was used for comparison.

The groups showed no differences in sexual distribution (*p* = 0.740). The preoperative assessment of facial weakness and hearing loss, together with morphology, did not produce significant group differences. The microsurgery group showed a higher occurrence of brainstem compression than the SRS group (*p* = 0.017).

The groups displayed no postoperative differences in facial weakness or hearing loss. Tumor status showed significant differences between the two groups (*p* < 0.001) as microsurgery patients experienced tumor shrinkage but SRS patients demonstrated tumor stability or slight growth. 

### **Age **group and intervention type

**Fig. 1:**
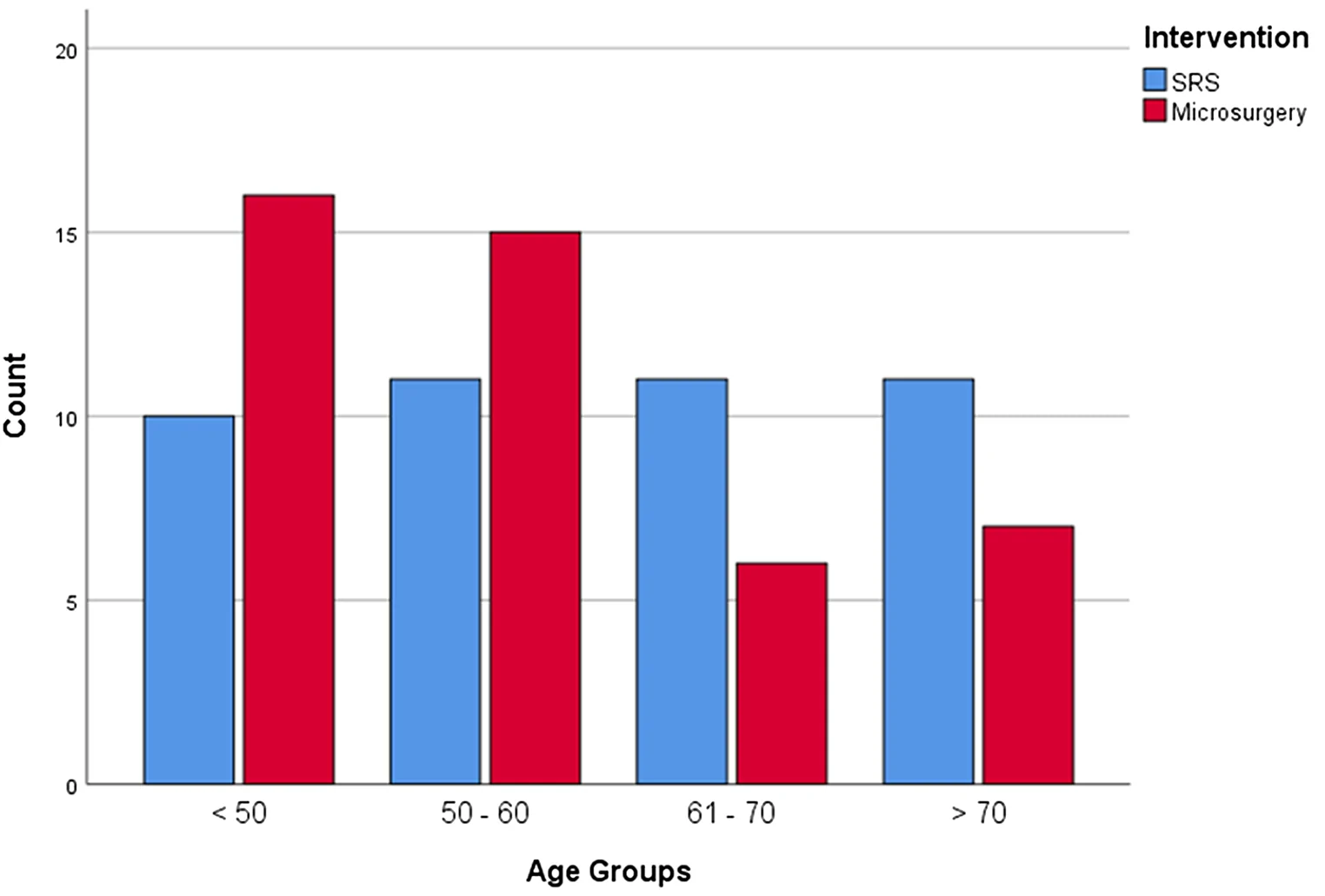
Comparison of treatment modalities across age groups in vestibular schwannoma patients. Note: No significant association was found between age group and intervention type (Pearson Chi-square = 4.35, df = 3, p = 0.226). The effect size wassmall (Cramer's V = 0.224). Therefore, age group distribution did not significantly differ between the SRS and Microsurgery groups

The age distribution of patients receiving interventions appears in Fig. 1. The Pearson Chi-square test result of 4.35 (df = 3, *p* = 0.226) with a Cramer’s V value of 0.224 revealed that age group did not influence treatment modality selection. Age group distribution did not affect treatment selection in a statistically significant manner.

### **Koos grade and intervention type**

**Fig. 2:**
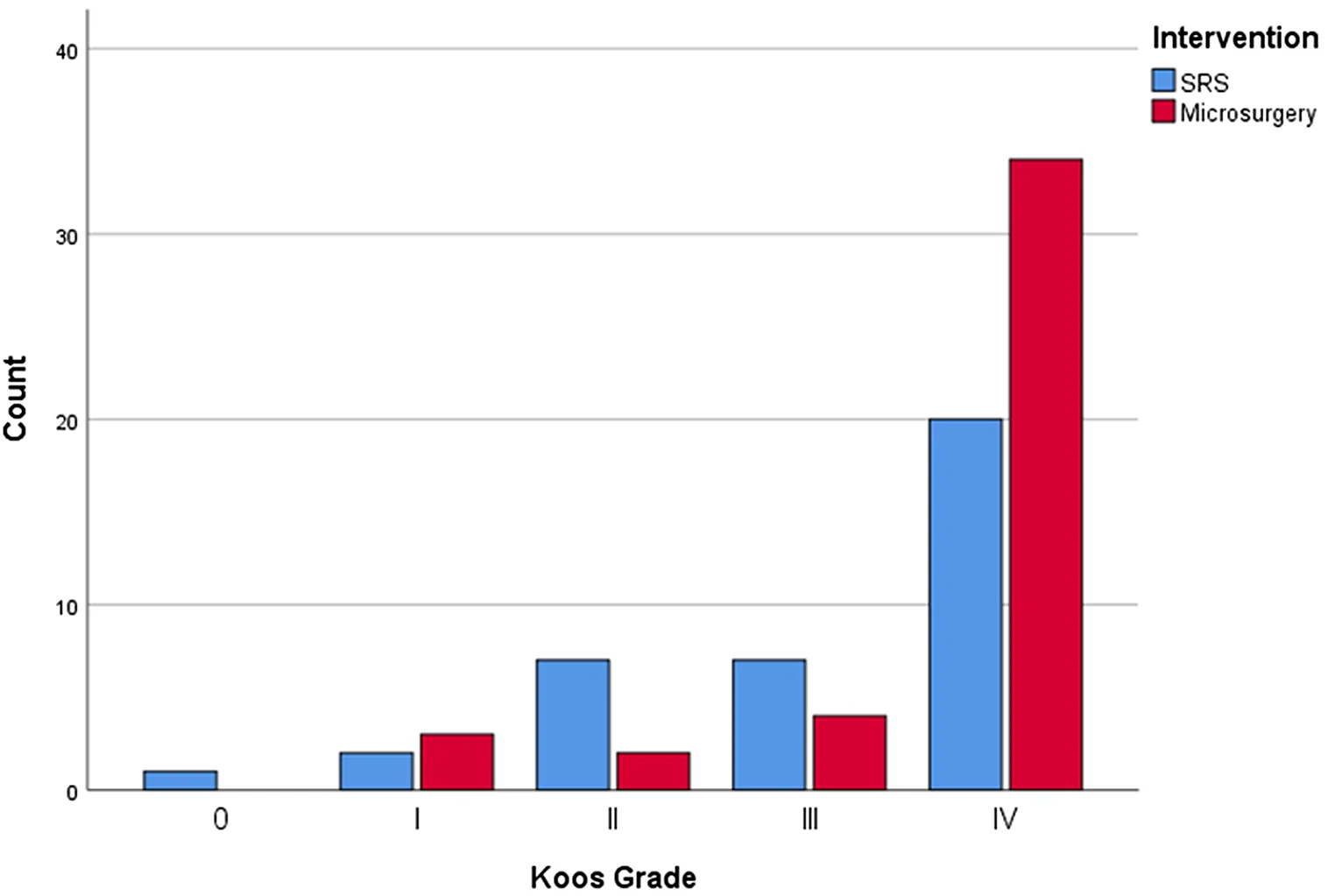
Treatment decision stratified by Koos grades of vestibular schwannoma. Note: The association between KOOS grade and intervention type was not statistically significant (Pearson Chi-square = 8.02, df = 4, p = 0.091).However, a trend toward significance was observed in the linear-by-linear association (p = 0.049), suggesting a possible ordinal relationship. The effectsize was small to moderate (Cramer's V = 0.317). Interpretation should be made with caution due to low expected counts in several cells

The bar chart in Fig. 2 shows how treatment choices varied based on Koos grade. Although the Pearson Chi-square test for intervention type versus Koos grade showed no significant association (*p* = 0.091, df = 4), the linear trend was statistically significant (*p* = 0.049), indicating that Koos grades influenced microsurgical treatment choices. The effect size was small to moderate (Cramer’s V = 0.317) so interpretation needs caution due to small expected frequencies in certain cells.

### **Tumor area and treatment effect**

The results from Table 4 reveal that treatment groups had different effects on tumor area percentages. Tumor area decreased by 76.04% in the microsurgery group but increased by 5.26% in the SRS group (*p* < 0.001, Mann–Whitney U test). The tumor area change data did not follow a normal distribution (Shapiro–Wilk *p* < 0.001) so a non-parametric test was employed.

The data presented in Table 5 shows how tumor areas evolved from preoperative to postoperative periods in separate groups. The microsurgery group experienced a significant decrease in tumor size after surgery (*p* < 0.001, Wilcoxon signed-rank test). The tumor area remained unchanged before and after SRS (*p* = 0.451).

### **Koos grade and clinical parameters**

The relationship between Koos grades and clinical parameters before and after surgery appears in Table 2. As expected by definition, pre-operative brainstem compression was observed in Koos Grade IV tumors. Hearing loss occurred more frequently at higher Koos grades (*p* = 0.030 and *p* = 0.020). The examination of facial weakness and morphology did not produce any significant findings.

**Table 1:** Demographics, pre- and post-operative clinical parameters between SRS and microsurgery for vestibular schwannoma treatment

Variable (Clinical Parameters)		All (*n* = 87)	SRS (*n* = 43)	Microsurgery (*n* = 44)	*P*-value
**Age**		55.87 ± 14.71	59.47 ± 13.58	52.36 ± 15.11	0.037
**Sex**	Male	40 (46.0%)	19 (21.8%)	21 (24.1%)	0.740
	Female	47 (54.0%)	24 (27.6%)	23 (26.4%)	
**Pre-operative**					
Facial Weakness	No	63	30	33	1.000
	Yes	21	10	11	
Hearing Loss	No	9	4	5	0.783
	Yes	73	36	37	
Brainstem Compression	No	26	17	9	0.017
	Yes	54	20	34	
Morphology	Cystic	13	4	9	0.164
	Solid	10	7	3	
	Mixed	21	9	12	
**Post-operative**					
Facial Weakness	No	49	27	22	0.121
	Yes	32	12	20	
Hearing Loss	No	8	4	4	0.881
	Yes	72	34	38	
Tumor Status	Shrink	45	5	40	< 0.001
	Stable	27	26	1	
	Growth	11	10	1	

Note: Age was not normally distributed based on the Shapiro–Wilk test (p = 0.018); therefore, the Mann–Whitney U test was used to compare age between groups. A significant difference was observed (U = 700.5,p = 0.037), with the SRS group being older on average. Other categorical variables Sex were compared using the Chi-square test

**Table 2:** Preoperative and postoperative clinical parameters vs. Koos grades

Clinical parameter	Category	Koos grade					*p*-value (χ²)	Cramer’s V
		Koos O	Koos I	Koos II	Koos III	Koos IV		
Pre-op facial weakness	No	1	5	7	10	36	0.259	0.257
	Yes	0	0	2	1	18		
Pre-op hearing loss	No	1	0	1	0	6	0.030	0.370
	Yes	0	5	8	11	46		
Pre-op radiology morphology	Cystic	0	-	0	0	13	0.431	0.266
	Solid	0	-	1	1	6		
	Mixed	1	-	1	4	15		
Post-op facial weakness	No	1	3	6	6	30	0.817	0.141
	Yes	0	1	3	5	23		
Post-op hearing loss	No	1	0	1	0	5	0.020	0.388
	Yes	0	4	7	11	48		
Post-op tumor status	Shrink	1	2	0	6	34	0.077	0.306
	Stable	0	2	4	4	14		
	Growth	0	0	3	1	5		

Among patients with Koos IV tumors (*n* = 54), 46 (85%) had hearing loss and 18 (33%) had facial weakness at presentation. Postoperatively, hearing loss was present in 48 patients (89%) and facial weakness in 23 (43%). Most Koos IV patients underwent microsurgical resection, with 34 demonstrating tumor shrinkage, 14 stability, and 5 growth at follow-up (Table 2).

**Table 3:** Exploratory Descriptive Statistics and Normality Test Results (*N* = 87)

Variable under test	Group	*N*	Median (IQR)	Shapiro–Wilk *p*-value
Preoperative Tumor Area (cm²)	All	85	3.89	< 0.001
	SRS	43	2.15 (0.89–4.59)	< 0.001
	Microsurgery	42	6.48 (1.75–11.58)	< 0.001
Postoperative Tumor Area (cm²)	All	82	1.96	< 0.001
	SRS	41	2.08 (1.28–4.29)	< 0.001
	Microsurgery	41	1.85 (0.56–4.83)	< 0.001
% Tumor Area Change	All	80	–25.11	< 0.001
	SRS	41	5.26 (–47.86 to − 9.20)	< 0.001
	Microsurgery	39	–76.04 (–94.6 to − 39.0)	< 0.001
Follow-up (months)	All	23	31.4	0.002
	SRS	3	31.40	0.216 (NS)
	Microsurgery	20	29.45	0.006

**Table 4:** Comparison of tumor area change between intervention groups

Intervention group	*N*	Median (% Change)	IQR (% Change)	Mann–Whitney U	Z-score	*p*-value
SRS	41	5.26	-38.66 to + 31.60	**93.000**	**-6.812**	**< 0.001**
Microsurgery	39	-76.04	-100.00 to -44.44			

Note: IQR = Interquartile Range, SD = Standard Deviation, Mann–Whitney U test was used due to non-normal distribution of tumor area change (Shapiro–Wilk p < 0.001 for both groups), and Positive values indicate an increase in tumor area; negative values indicate a reduction

**Table 5:** Pre- and postoperative tumor area comparison within intervention groups

Procedure under test	*n*	Tumor area median (IQR)		Z-value	*p*-value
		Pre-op	Post-op		
SRS	41	2.15 (0.98–4.68) cm²	2.08 (1.33–4.34) cm²	-0.754	0.451
Microsurgery	39	6.48 (3.21–13.04) cm²	1.85 (0.00–4.27) cm²	-5.344	< 0.001

Note: Wilcoxon Signed-Ranks Test

Tables 3, 4 and 5 summarize the paired pre- and post-treatment data for tumor size and tumor area. Microsurgery consistently achieved marked reductions, with a median tumor area decrease of 76%, while SRS maintained stability with only modest change (5%).

## Discussion

In this single-institution cohort, we observed distinct radiographic and clinical patterns in the management of vestibular schwannomas treated with either microsurgical resection or SRS. Treatment decisions, including radiosurgical planning, were made within a multidisciplinary clinical framework that incorporated input from radiation oncology, in accordance with established institutional protocols. Patients in our cohort were frequently referred at advanced stages of disease, as reflected by the high proportion of Koos grade IV tumors. This likely explains the relatively high rates of preoperative facial weakness. The novelty of our analysis lies in the use of pre- and post-treatment data to quantify both tumor response and functional outcomes. Rather than relying solely on long-term control rates, our study demonstrated how each treatment directly alters tumor size and area in individual patients. Given the inherent limitations of a non-randomized retrospective design, including treatment allocation driven by baseline tumor characteristics, this study is intended as an exploratory, hypothesis-generating analysis rather than a definitive comparative effectiveness assessment. Surgical resection provided immediate and substantial reduction, whereas SRS maintained radiographic stability with similarly low rates of new neurological deficits.

When examining tumor response in detail, microsurgery achieved a median tumor area reduction of approximately 76%, whereas SRS was associated with relative stability or modest growth of about 5% following treatment (Tables 3, 4 and 5). These findings are consistent with outcomes reported in recent series and meta-analyses of vestibular schwannoma management [7, 12, 13]. Our paired comparison of clinical parameters before and after intervention (Tables 1 and 2) demonstrated that marked volume reduction following microsurgery did not translate to higher rates of facial palsy or hearing loss. Our clinical outcomes suggest that contemporary surgical techniques, supported by advances in intraoperative monitoring and perioperative care, can achieve substantial tumor debulking without a disproportionate increase in morbidity, though these findings should be interpreted in the context of the study’s retrospective design and limited confounder adjustment.

Conversely, the stability achieved with SRS was functionally sufficient in most patients, reinforcing its role as a minimally invasive alternative for older individuals or those with comorbidities. Although our study is limited in its ability to account for clinical acumen, nuanced scenarios, and temporary palsies, our evidence challenges the historical perception that aggressive resection invariably compromises cranial nerve outcomes and supports the role of surgery as a safe and effective intervention when indicated [14–18]. 

The Koos classification system remains an essential framework in treatment planning for vestibular schwannomas [19, 20]. In our cohort, Koos grade correlated strongly with both brainstem compression and hearing loss, and a significant linear trend was observed toward surgical management with higher grades. The Koos IV subset, defined by brainstem compression, highlights the clinical challenges of advanced tumors. Nearly all patients in this group were directed toward surgical resection, underscoring the practicality and reliability of the Koos system in guiding management decisions. Despite higher baseline morbidity, postoperative outcomes in Koos IV patients did not show a disproportionate increase in facial nerve or hearing deficits compared with lower-grade tumors. These findings are consistent with prior work demonstrating the reproducibility of the Koos system across institutions and underscore its ongoing relevance in contemporary vestibular schwannoma management [21, 22]. 

This study has several limitations inherent to its retrospective design. Detailed radiosurgical parameters, including dose and fractionation, as well as standardized follow-up intervals and regrowth rates following subtotal resection, were not consistently available across all patients, limiting the ability to perform granular subgroup analyses. Follow-up duration was heterogeneous across the cohort, with longitudinal imaging available in a subset of patients. Given the variability and limited duration of follow-up, long-term tumor control and durability of outcomes could not be reliably assessed. Functional outcomes, particularly facial nerve function, were reported in a categorical manner due to inconsistent use of standardized grading systems such as the House–Brackmann classification, which may reduce comparability with existing literature.

In addition, the extent of resection was not uniformly documented using standardized criteria, limiting interpretation of postoperative tumor reduction and precluding analysis of outcomes based on degree of tumor removal. Extent of resection was characterized using area-based percentage rather than volumetric assessment, and the report median reduction should not be interpreted as a formal resection extent classification. Without volumetric data, standard categories such as gross total, near-total, or subtotal resection could not be reliably applied to individual cases.

Information regarding prior observation before intervention was also not systematically available, restricting assessment of pre-treatment tumor growth dynamics. Tumor size was assessed using area-based measurements derived from radiological reports rather than volumetric analysis; although volumetric assessment is increasingly considered standard, the use of area-based measurements allowed for consistent data extraction across the cohort. In particular, area-based assessment is susceptible to misinterpretation in the SRS group, where transient post-radiation enlargement is well recognized and can confound short-term radiographic response. Volumetric analysis with standardized imaging intervals would be required to more precisely characterize treatment response following SRS. Additionally, the timing of postoperative imaging was not standardized across the cohort, varying based on clinical follow-up practices across the study period. Tumor status was recorded as shrinkage, stability, or growth based on radiologist interpretation at the time of imaging rather than a predefined quantitative threshold, which should be considered when interpreting the reported rates of tumor shrinkage and stability.

Furthermore, the study was not designed to compare outcomes between conservative and aggressive resection strategies within the surgical cohort, as extent of resection and intraoperative decision-making parameters—including electrophysiologic monitoring thresholds and criteria guiding the extent of tumor removal—were not consistently recorded. These factors limit the ability to fully evaluate surgical decision-making and its impact on outcomes.

Collectively, these limitations reflect the variability in documentation inherent to retrospective analyses and should be considered when interpreting the findings. Future prospective studies with standardized reporting of surgical, radiological, and functional parameters are warranted to validate and expand upon these results.

The volumetric differences identified in this study highlight the need for standardized, objective approaches to tumor measurement across treatment modalities. Future investigations should validate these findings in larger, multi-institutional cohorts with long-term follow-up and uniform volumetric assessment protocols. Advanced analytic tools such as radiomics and machine learning could enhance this process by automating tumor segmentation and generating predictive models for cranial nerve outcomes. Integration of molecular profiling, including gene sequencing, may provide complementary biological markers of tumor behavior that refine risk stratification beyond size and Koos grade. Finally, prospective studies incorporating quantitative audiometric data, patient-reported outcomes, and cost-effectiveness analyses will be essential to capture the full clinical impact of treatment choices and guide evidence-based counseling. This study should be interpreted as an exploratory, hypothesis-generating analysis rather than a definitive comparative effectiveness study.

## Conclusion

This study provides a head-to-head comparison of microsurgical resection and SRS for vestibular schwannoma using paired pre- and post-treatment data. In this cohort, microsurgery achieved immediate and substantial tumor reduction without an observed increase in cranial nerve morbidity, while SRS maintained radiographic stability with similarly low risk. These results challenge the historical assumption that aggressive resection inevitably compromises function and highlight the complementary roles of surgery and radiosurgery in modern practice.

The correlation between Koos grade and treatment selection underscores the ongoing value of this classification system in guiding management. Although limited by sample size in a retrospective design, our findings emphasize the importance of volumetric analysis and paired outcome measures in clarifying treatment effects. Larger, prospective studies incorporating standardized volumetric metrics, molecular profiling, and patient-reported outcomes will be essential to refine treatment decision making, and to improve evidence-based counseling for patients with vestibular schwannoma.

## Data Availability

No datasets were generated or analysed during the current study.
